# A comprehensive model and computational methods to improve Situation Awareness in Intelligence scenarios

**DOI:** 10.1007/s10489-021-02673-z

**Published:** 2021-08-01

**Authors:** Angelo Gaeta, Vincenzo Loia, Francesco Orciuoli

**Affiliations:** Dipartimento di Scienze Aziendali - Management, Innovation Systems (DISA-MIS), Università degli Studi di Salerno, Via Giovanni Paolo II, 132, 84084 Fisciano, Italy

**Keywords:** Intelligence analysis, GrC, SA

## Abstract

This paper presents a comprehensive model for representing and reasoning on situations to support decision makers in Intelligence analysis activities. The main result presented in the paper stems from a work of refinement and abstraction of previous results of the authors related to the use of Situation Awareness and Granular Computing for the development of analysis methods and techniques to support Intelligence. This work made it possible to derive the characteristics of the model from previous case studies and applications with real data, and to link the reasoning techniques to concrete approaches used by intelligence analysts such as, for example, the Structured Analytic Techniques. The model allows to represent an operational situation according to three complementary perspectives: descriptive, relational and behavioral. These three perspectives are instantiated on the basis of the principles and methods of Granular Computing, mainly based on the theories of fuzzy and rough sets, and with the help of further structures such as graphs. As regards the reasoning on the situations thus represented, the paper presents four methods with related case studies and applications validated on real data.

## Introduction

According to NATO,^1^ Intelligence can be defined as: *“the product resulting from the directed collection and processing of information regarding the environment and the capabilities and intentions of actors, in order to identify threats and offer opportunities for exploitation by decision-makers*”. Closely connected to the definition of Intelligence, there is the one of Intelligence analysis which consists of several cognitive methods that are commonly referred to as analytic tradecraft [32] and that can be subjects to errors and biases [23]. Intelligence analysis is usually executed within an Intelligence Cycle which is the process of developing raw information into finished Intelligence for policymakers to use in decision-making and action.

In the concluding thoughts of [48], authors state that “*the best intelligence analysis derives from the right combination of art and science. The art of Intelligence may be the same today as it was 2,000 years ago. What is different now, however, is the necessity of getting much better much faster at the science of the tradecraft, which is centered on data. Analysts must have the tools they need to deal with massive amounts of information that enable them to close Intelligence gaps and enable better operational outcomes at the speed of data.”* The emphasis is therefore placed on a correct combination of art (i.e., methods and techniques of Intelligence analysis) and science which is currently based on data driven technologies. However, problems can arise when this combination is not well balanced and one of the two elements (art or science) takes over the other. An example of Intelligence failure is reported in [48] where authors emphasize how *“in a more data-oriented era, it is increasingly possible to draw Intelligence of value from the data in aggregate (temporal and geospatial behavior patterns, for example). This can result in an ironic dilemma in which there is too much data for humans to search effectively for needles, yet not enough accessible data from which to draw and validate useful intelligence”*. It is clear that problems related to cognitive errors and biases, as well as information overload, lead to risks associated with Intelligence analysis activities. This challenge requires models, methods, techniques and tools to reduce errors and minimize biases when human operators need to make decisions in mission-critical scenarios.

For this purpose, the construct of Situation Awareness (SA) can support decision makers when they need to acquire an improved awareness of operational and mission critical situations. SA is being aware of what is happening around you and understanding what that information means to you now and in the future. With the support of software systems, it can be obtained by fusing individual pieces of information (e.g. sensor data) and interpreted in an abstract, domain-relevant concept, called “situation”. SA has been proved to reduce some sources of error [11]. However, SA has its demons that are factors or causes dampening the awareness of situations [14]. Some of these demons, such as data overload and complexity creep, can be mitigated using an information processing paradigm such as Granular Computing (GrC) that allows data and information to be grouped according to different perspectives (i.e., granulation), and organize information by means of levels, hierarchies and granular structures.

The authors of this paper studied a systematic integration of SA and GrC in previous works [7, 30]. In this paper, the main contribution refers to the definition of a comprehensive model to represent and reason on situations with the aim of supporting Intelligence analysis activities for decision-making. The study that led us to these results originated from a twofold intuition: i) to revise some analytical tradecraft techniques within a cognitive framework aimed at increasing the situational awareness of decision makers and, ii) to leverage on granular information processing approaches that allow to represent and reason on situations or on their elements at different levels of abstraction.

Currently, in relation to decision making, two main research trends can be observed in this sector: the first concerns the study of autonomous systems [45] characterized by decision-making autonomy of software agents, the second focuses on human-centered systems [48] which are characterized by providing support to the human decision maker. In the first case, humans are outside the decision-making process (Human out of the loop) while in the second they are part of this process (Human in the loop).

The results presented in this paper relate to the second trend mentioned above: the development of human-centered systems aimed at decision making for critical sectors and applications such as safety and security.

### Background and related works

This section reports some background information and related works on SA and GrC.

#### Situation awareness

Endsley [12] defines SA as “*the perception of elements in the environment within a volume of time and space, the comprehension of their meaning, and the projection of their status in the near future*”. SA is a cognitive construct devoted to support humans and agents in taking informed decisions. SA helps to interpret and understand information in the context of a larger concept called *situation*, which is an abstract state of affairs related to specific applications.

The Endlsey’s model of SA [14] consists of three levels devoted to support: *i)* the perception of the elements of the environment (level 1), *ii)* the comprehension of the current situation, which refers to the understanding of what data and cues perceived mean in relation to goals and objectives (level 2), and *iii)* projection of the situation in the near future (level 3).

The model is iterative, with the comprehension driving the search for new data and new data coming together to feed understanding, and it combines data-driven and goal-driven information processing. In fact, external and internal factors such as goals, mental models, attention, working memory, expectations play a pivotal role in SA [12, 14].

SA immediately found concrete applications in sectors such as military [16], air control and aviation [10, 42]. With respect to Intelligence analysis and cycle, in [38] it is emphasized how the Intelligence Cycle has a long history and, to keep the current cycle and obtain value from its execution, it will need to be augmented with SA, explanatory value, prediction, and strategic notice. This is true regardless of the originating source of information (i.e., Open-Source, Human, Geo spatial).

In order to enhance the Intelligence Cycle, we can refer to the results of [47]. In [47], authors develop an information security risk management process based on SA. As an intermediate result, authors produce a mapping between the phases of Intelligence Cycle and Endsley’ SA model. The model, that is referred to as US National Security Intelligence Enterprise (USNSIE), divides an Intelligence Cycle into the phases executed within an Intelligence community, from Collection to Dissemination, and the phases pertaining the information producers (i.e., the decision maker). Phases related to Requirements elicitation, Planning and Direction relate to goals and objectives. To execute these activities, Endsley proposes the adoption of Goal-Directed Task Analysis (GDTA). GDTA is also used in our approach to modeling and reasoning on situations, as will be detailed in Section 2.

#### Granular computing and three-way decisions

As research area, GrC [37, 52, 53] takes its origin from Zadeh intuition, who defined a granule as clump of objects *drawn together by indistinguishability, similarity, proximity, and function* [67]. A granule is an elementary information that can be constructed with a process called granulation. The specific way by which this process is executed as well as the nature of an information granule differ on the basis of the formal setting adopted for GrC e.g., fuzzy sets [36], rough sets [34], orthopairs [4], intervals [68].

Yao in [59] presents a triarchic theory of granular computing that integrates three important perspectives, namely: philosophy of structured thinking, methodology of structured problem solving, and mechanism of structured information processing. The main point discussed is the capability of GrC to exploit useful structures to enforce multi-level and multi-view understanding. These structures are called granular structures and consists of basic chunks of information, namely information granules. Granular structures are constructed and interpreted following the principles of multi-view and multi-level [54] emphasizing comprehension and representation of information, respectively, from multiple perspectives and multiple levels of abstraction.

The possibility offered by GrC to reason with multi-level and multi-view structures allows to reinforce the phases of an Intelligence Cycle. In this paper, we mainly focus on the information processing perspective of the GrC and on its added values for the SA. We refer to [30] for an overview of GrC techniques and methods that can enforce SA while, in the following, we report some related works on the adoption of GrC to support Intelligence analysis.

In line with the triarchich theory of GrC, Wang in [46] investigates the combination of GrC and Cognitive Computing [27] and defines a Data-Driven Granular Cognitive Computing (DGCC) model that combines data-driven bottom-up information processing with a top-down cognition mechanism based on the global precedence law [21]. The approach proposed by Wang shares with us the intuition of balancing bottom-up and top-down information processing to enforce decision making. DGCC differs from our vision of SA based on GrC for the different cognitive framework. We base our vision on SA and the top-down information processing relies on the GDTA structure. This choice allows us to be more focused on specific sectors and applications where SA has been proved to be a competitive advantage.

Strictly related to GrC, cognitive computing and decision making, is the Three-Way Decisions (3WD) theory. A model based on 3WD supports decision-making processes based on a trisecting-and-acting model [58]. This type of model is based on two tasks: the division of the universal set into three pairwise disjoint regions and the definition of actions or strategies to act upon the objects of the three regions. The three regions are, usually, referred to as positive (POS), negative (NEG) and boundary (BND). This model has been generalized into a trisecting-acting-outcome (TAO) model [60], thus taking into consideration the outcome. In brief, in the TAO model, a third aspect, related to the evaluation of the effectiveness of both trisection result and strategy, is introduced.

In [58], Yao clearly exposes cognitive biases and advantages of using 3WD in several domains and in his recent work [61] explores the geometric and graphical representations, as well as the semantic interpretations of several structures that can be built with 3WD. As we will see in the following, 3WD is a pillar of our works. We use 3WD for rapid decision making in several phases of Intelligence analysis and also as a reasoning mechanism to classify situations.

### Organization of the paper

The paper is organized as follows. Section 2 presents the vision underlying the integration between SA and GrC. Section 3 describes the model to represent a situation according to three complementary perspectives: descriptive, relational and behavioral. Section 4 and related subsections provide an overview of the techniques to reason on situations represented with the model. Section 5 presents case studies and applications and, lastly, Section 6 draws conclusions with a discussion on open issues and future developments.

## The vision: situation awareness based on granular computing

**Fig. 1:**
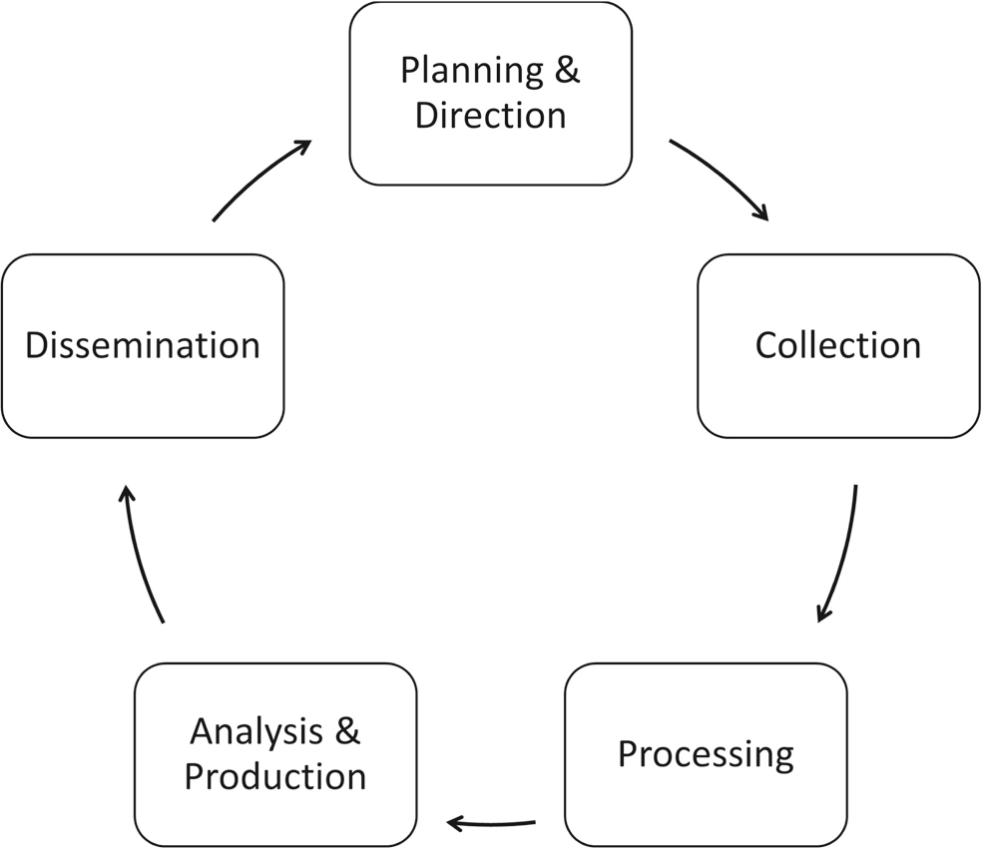
An Intelligence Cycle

As introduced in Section 1, the main challenge afforded in this paper is supporting decision-making processes for Intelligence analysis. Usually, Intelligence analysis activities are executed along an Intelligence Cycle such as the one of Fig. 1 showing the five phases of an Intelligence Cycle. The cycle starts with the phase of Planning and Direction, which is aimed at identifying objectives and requirements, and planning the information gathering activities. The cycle continues with the Collection and Processing of data and information, and their Analysis to Produce new information to be Disseminated within the communities of interests.

To execute an Intelligence Cycle, a vision based on SA and GrC is proposed in this paper. The paradigm underlying the vision is that of SA, with particular reference to the information processing perspective of the Endsley’s Model [70] in which situations are handled by perceiving, abstracting/interpreting and projecting (in the near future) data produced by the environment of interest. The main consideration conducting to the aforementioned choice is that high levels of situation awareness lead to better decisions especially if such decisions have to be taken in complex and critical contexts [13].

However, to gain benefits from SA, situations need to be represented in a computational model allowing to reason and make decision on them.

To fulfill the aforementioned need, GrC seemed to be the most flexible set of paradigms to formally deal with data at different levels of abstraction. In such a scenario, the work [30] analyzes the aforementioned capabilities in order to emphasize the benefits of granular computing when considered as an enabler for situation awareness solutions. The advantages of granular computing are multiple: the possibility to adopt a plethora of formal settings (e.g., Rough Set Theory, Fuzzy Logic, etc.), its flexibility (as mentioned before) to be deployed for solving a wide range of problems, its capability to build structures as the human brain does (cognitive approach), to allow humans to be included in the loop to improve the processing, to co-operate with automatic agents, etc.

More in detail, in the proposed vision, situations are mainly represented by means of *granular structures* constructed through the execution of *granulation operations*. The problem space is, firstly, defined by gathering data represented by objects in the environment of interest. Each object is described by one or more values associated to a given set of features. Granular structures mainly enable a *descriptive* perspective of a situation. Two optional perspectives can be added to the first one: *relational* and *behavioral* perspectives. The *relational* one can be enabled by structures representing relations among objects and also properties of such relations. Moreover, the *behavioral* one can be defined by means of techniques useful to analyze and abstract the actions of the aforementioned objects within the environment. Now, a good conceptual overview of how a situation should be computationally represented is obtained.

**Fig. 2:**
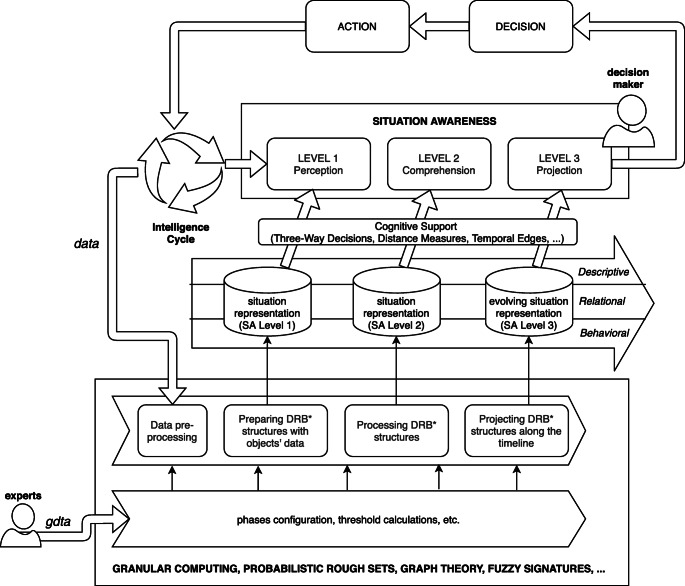
Proposed vision based on **D** escriptive, **R** elational and **B** ehavioral perspectives of situation representations

Figure 2 offers a sketch of the proposed vision. In particular, the decision maker’s SA is enforced along the three SA levels by three different levels of situation representations which are connected. SA level 1 is supported by the representation of situation accomplished by structuring objects data related to descriptive, relational and behavioral perspectives. At this level, there is no further abstraction, data are gathered, pre-processed and opportunely organized within suitable structures. SA level 2 is improved by processing the situation representation offered at level 1 through the application of specific operators able to provide higher levels of abstraction. In particular, the situation representation at SA level 2 consists of the aforementioned granular structures for the descriptive perspective, measures expressing the relational centrality of each object in the environment and, lastly, vectors summarizing the behavior of the objects. SA level 3 is supported by a more complex situation representation in which temporal and evolution aspects of situations have been considered. In the proposed vision, human operators are provided with additional cognitive tools, through which reasoning on the situation representations, to support the achievement of suitable SA. Such cognitive tools, e.g., Three-Way Decisions, Distance Measures, are useful to decrease the cognitive load of the decision maker while she is trying to achieve her goal, i.e., to support her decisions and, consequently, her actions. The situation representations at the three SA levels are constructed by means of processes realized by employing formal methods and theories (e.g., granular computing, probabilistic rough sets, graph theory, fuzzy signatures, etc.) and configured also through the analysis and design phase mostly accomplished by GDTA [15] with the intervention of human experts. GDTA is a form of cognitive task analysis and focuses on the goals the human operator must achieve and the information requirements that are needed to make appropriate decisions. Information is, step-by-step, decomposed until reaching finer elements that cannot be further decomposed. It is important to underline that GDTA focuses on dynamic information requirements rather than static system knowledge, i.e. the approach considers the information, necessary to perform a specific task well, that needs to be acquired and analyzed by the operator in a certain domain during the execution of that task. The needs for this information are called SA requirements. GDTA is useful to catch aspects like, for instance, relevant features of the considered objects, situations of interests, decisions to be made and associate these aspects to the SA requirements and, finally, GDTA provides requirements for the granulation process. A GDTA provides information requirements on SA at all the three levels: perception, comprehension and projections. This allows to identify the correct subset of attributes (such as velocity, distance, altitude) and proper binary relations (such as equivalence, proximity, dominance) between objects of the environment to support a correct granulation. In other words, GDTA is the tool allowing a meaningful representation of a situation via a granular structure.

## The descriptive, relational and behavioral model of situation

To implement the vision described in Section 2, it is necessary to define a situational model that must be formal, explicit and actionable. With this last term, we refer to the fact that the model has to support human operator actions and rapid decision making avoiding a traditional “black-box” machine learning approach that leaves humans out of the loop. The challenges related to situation modeling have been discussed in our previous works such as [7, 30] and [8]. In brief, as Endsley emphasizes in [11], most of human errors concern the difficulty in perceiving and comprehending situations. This can be smoothed if we are able to define a computational model allowing to reason on situations.

In literature, there are several approaches to model situations. An interesting work dealing with these problems is the review [63] that describes and compares specification-based and learning-based techniques. The former includes the adoption of fuzzy logic [66], ontologies [28], evidence theory, [41], situation theory [9] and combinations of them, such as [25]. Specification-based techniques have the advantage of representing explicitly and formally a situation with the possibility of making inference on those representations but, usually, they are not so flexible as to adapt to changes without substantial modifications. Learning-based techniques consist of approaches such as Naive Bayes, hidden Markov model, neural networks and other methods that are able to learn complex associations between situations and sensor data but, on the other side, do not provide a formal and explicit model of the situations with the risk of leaving the human operator out of the loop. The combination of specification and learning-based techniques is required in concrete scenarios of operational situations.

**Fig. 3:**
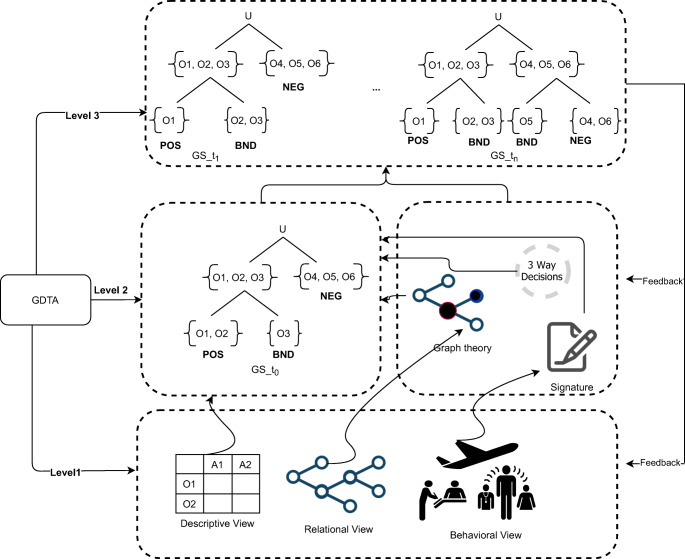
The DRB model

The Descriptive, Relational and Behavioral (DRB) model of situation presented in this section has been elaborated starting from the results reported in [8]. Following a specification-based approach and guided by the principle of maintaining the human in the loop, in [8] the problem of situation modeling has been investigated proposing an approach based on a lattice of partitions, where a partition represents a set of objects/elements that are fused according to GDTA requirements. This approach, allowing to represent a situation in accordance with the information requirements of the GDTA, is what we refer to as the descriptive perspective of the situation. While this may be enough to reason on situations in many cases, some applications need a more comprehensive situational model. For example, to remain in the domain of Intelligence analysis, we can refer to intentional attacks on large-scale infrastructures or the analysis of situations involving the behavior of human or software agents. In these cases it is necessary to reinforce the descriptive perspective of a situation with elements and structures that allow to represent the relationships between the objects of an environment and to compute their behavior. For this reason, in this paper, we propose a model that integrates the three perspectives: descriptive, relational and behavioral. Let us discuss the model with the support of Fig. 3.

As mentioned, a situation can be defined as an abstract state of affairs related to a specific application. This state of affairs can be modeled according to three different perspectives: a) descriptive, which is aimed at representing the objects of an environment and the observations that are measured on these objects, b) relational, which is aimed at modeling the relationships between the objects of an environment, c) behavioral, which focuses on the actions and behaviors of objects in an environment. To improve SA, each one of these three perspectives has to be computationally modeled with a set of methods and techniques that are such to enforce SA at Level 1, 2 and 3.

Let us define a Situation Model, *SitMod*, as a triple:

1 $$  SitMod = \langle DesMod, RelMod, BeaMod \rangle $$

consisting of a descriptive model, *DesMod*, a relational model, *RelMod*, and a behavioral model, *BeaMod*. (1) finds different determinations in relation to the specific phase of SA it has to model, i.e.: perception, comprehension or projection, on the basis of the adopted concrete formal setting. A modeling can be more or less rich depending on whether the elements of (1) are complete or not. It is important to highlight that the descriptive component of (1) is the fundamental pillar of our modeling approach. This component can be enriched with one or both of the other two components on the basis of the specific application.

Let us discuss a concrete contextualization of (1) to the three levels of SA on the basis of GrC and computational intelligence techniques.

### SA Level 1: Perception

For the perception level, (1) consists of an information system, a graph and a multi-dimensional data structure object-action-target-resource: *SitMod*_*L*1_ = 〈*IS*,*G*,〈*Obj*,*Act*,*Tar*,*Res*〉〉.

An information system [35], *IS*, is a data table whose columns are labeled by attributes, rows are labeled by objects of interest and entries of the table are attribute values. Formally: *IS* = (*U*,*A*) where *U* is a non empty universe of objects and *A* is a set of attributes. With every attribute *a* ∈ *A* we associate a set *V*_*a*_ of its values, called the domain of *a*. If the attribute set includes both condition, *C*, and decision, *D*, attributes then *A* = *C* ∪ *D* and *IS* is called decision system. *IS* allows to describe the objects of an environments with respect to their qualities (i.e., the attributes of *A*) and observations (i.e., values from *V*_*a*_).

Graphs are structures used in the relational perspective. A graph is defined as a pair *G* = (*V*,*E*), where *V* is a set of nodes and *E* = {(*x*,*y*)|(*x*,*y*) ∈ *V*^2^*andx*≠*y*} is a set of edges. In our case, $V \subseteq U$ are the objects of the environment and *e* ∈ *E* denotes a relation between two objects.

Lastly, to model the behavioral perspective, it is adopted the approach proposed in [50] where Yager and Reformat use a 3D data structure to model user activities on social networks. Borrowing their intuition, we use a multi-dimensional structure 〈*Obj*,*Act*,*Tar*,*Res*〉 to represent the action of the objects of the environment where $Obj \subseteq U$ are objects of the universe, *Act* denotes a set of actions, *Tar* denotes a set of target of an action and *Res* denotes a set of resources used in the action. If an object, *o*_*i*_, executes an action, *a*_*j*_, towards a target, *t*_*k*_, using a resource, *r*_*l*_, then a point 〈*o*_*i*_,*a*_*j*_,*t*_*k*_.*r*_*l*_〉 in 〈*Obj*,*Act*,*Tar*,*Res*〉 is marked.

### SA Level 2: Comprehension

The three structures above described allow to represent a situation in terms of objects of the environment, their relations and behaviors supporting the perception of elements related to occurring situations. These structures are building blocks that can be further elaborated with the support of information processes, such as granulation, to derive other structures for the comprehension phase of SA. (1) is determined for the comprehension level as follows: *SitMod*_*L*2_ = 〈*GS*,*NI*,*OS*〉 where *GS* is a granular structure, *NI* is a set of network indicators and *OS* is a set of fuzzy signatures [50]. Let us defines these concepts and discuss their adoption for our purposes.

A granular structure can be defined as a mathematical structure of the collection of information granules, in which the inner structure of each granule is visible and the interactions among granules are detected by the visible structures [40]. Given an universe *U* and a binary relation *R* over *U*, a granular structure *GS* can be defined as follows:

2 $$  GS(R) = (g_{R}(o_{1}), g_{R}(o_{2}), ..., g_{R}(o_{n})) $$

where

3 $$  g_{R}(o_{i}) = \frac{p_{i1}}{o_{1}} + \frac{p_{i2}}{o_{2}} + ... + \frac{p_{in}}{o_{n}} $$

is the granule induced by *o*_*i*_ on the basis of *R* and + refers to union. *p*_*ij*_ is a membership for the *j* − *th* elements and *p*_*ii*_ = 1. If *p*_*ij*_ ∈{0,1} then (2) refers to a crisp granular structure and (3) to a crisp granule constructed, for instance, with the equivalence relation of Rough Set [34]. If *p*_*ij*_ ∈ [0,1] then (2) refers to a fuzzy granular structure and (3) to a fuzzy granule constructed, for instance, with a similarity relation. (3), in general, denotes an information granule constructed on the basis of a binary relation *R*. Depending on the specific relation, (3) includes all the objects that are similar, indistinguishable, proximal, etc. to *o*_*i*_. Since *R* can be defined on different subsets of attributes, a *GS*(*R*_*B*_) induced by *R*_*B*_ where *B* ⊂ *A* is a refinement of *GS*(*R*). The refinement of a *GS* can lead to a lattice of partitions such as that one shown in the middle of Fig. 3 labeled with $GS_{t_{0}}$.

What is the value of *GS* for SA? It lies in the capability of fusing the objects of an environment according to the requirements of SA Level 2 and in the possibility of refining and coarsening a *GS*. The different requirements for information fusion can be implemented with granulation processes based on different binary relations. As mentioned, these benefits come from the adoption of a GDTA structure that gives information requirements on SA at all the three levels. Furthermore, the adoption of *GS* for the descriptive perspective supports reduction of SA errors [11]. Several and common errors at the perception level of SA relate to difficulty to perceive and observe data. These errors can be reduced with a proper granulation process that follows GDTA requirements. An effort is required to assure flexibility to accommodate GDTA requirements but, if granulated and properly organized, data becomes more easy to be perceived and comprehended.

As highlighted in Fig. 3, at the SA level 2, the *GS* is mandatory to improve the comprehension of a situation. The *GS*, however, may be not always sufficient (for instance, when the objects have non-trivial behaviors - or relations - which are relevant for the analysis goal) and can be enriched with other structures and indicators, such as graph theory indicators and signatures, and also with the 3WD theory that, at this stage, can support classification of the situations.

A *GS* gives a snapshot of the current situations informing decision makers on objects that are similar, geographically proximal, and so on. In several scenarios, however, this information has to be enriched with a relational perspective consisting of indicators and measures relating to the connection among objects. For example, scenarios concerning large scale systems and critical infrastructures, where measures such as influence or centrality of nodes are required to comprehend the situation. This motivates the adoption at this level of a set of network indicators, *NI*, evaluated from the graph structure of level 1. For instance, a network measure such as the the Katz centrality [26], typically used for estimating the relative influence of actors in a social network, can be used as an evaluation function (or in combination with other functions) to granulate a large-scale system on the basis of the criticality and influence of the objects / nodes. In this way, the centrality measure enriches the descriptive model of a *GS* by highlighting in the *GS* information granules or single objects of a large-scale system that are most critical in a particular situation.

Lastly, the comprehension of the situation improves if the decision maker also has at her/his disposal a modeling of the objects’ behavior. The behavioral perspective is based on the object fuzzy signature, *OS*, developed following the approach of user fuzzy signature presented in [50]. In brief, in the behavioral perspective, a fuzzy signature for an object *o*_*i*_ is a fuzzy relation between the fuzzy sets $\overbrace {Act}$, $\overbrace {Tar}$ and $\overbrace {Res}$, that can be constructed starting from the multidimensional structure < *Obj*,*Act*,*Tar*,*Res* >. Formally:

4 $$  OS_{o_{i}}(a, t, r) = \overbrace{Act_{o_{i}}}(a) \times \overbrace{Tar_{o_{i}}}(t) \times \overbrace{Res_{o_{i}}}(r) $$

and for a specific action, *a*_*j*_, on a specific target, *t*_*k*_, using the specific resource, *r*_*l*_, the value is given by

5 $$  OS_{o_{i}}(a_{j}, t_{k}, r_{l}) = min \lbrace \overbrace{Act_{o_{i}}}(a_{j}), \overbrace{Tar_{o_{i}}}(t_{j}) \overbrace{Res_{o_{i}}}(r_{l}) \rbrace $$

with high values indicating a preference for the action *a*_*j*_ towards the target *t*_*j*_ using the resource *r*_*l*_. This information support comprehension in the same way as described for the network indicators, given that it allows to create behavioral profiles of the objects that can enrich the *GS*.

**Fig. 4:**
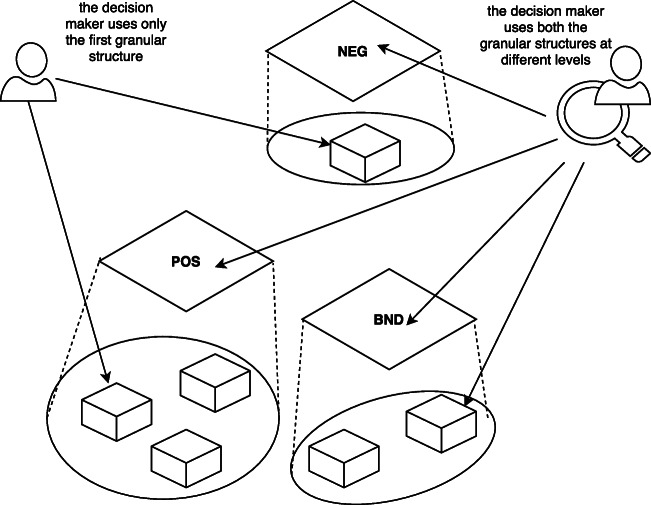
Individual and combined usage of granular structures

The three structures described, *GS*, *NI* and *OS*, can be used to comprehend a situation. However, an important part of comprehension is related to the classification of an operational situation. To support the classification, we employ 3WD theory. Yao in [60] discusses the connections between 3WD and GrC. In general, it can be used to divide an universal set in three disjoint regions. Let *U* be our universal set, *u* ∈ *U* is an object and *v*(*u*) an evaluation function. Let us define two thresholds, 1 ≥ *α* > *β* ≥ 0. With 3WD the universe can be divided in three subsets:

6 $$  \begin{aligned} POS(U)= \lbrace u \in U | v(u) \geq \alpha \rbrace \\ BND(U)= \lbrace u \in U | \beta < v(u) < \alpha \rbrace \\ NEG(U)= \lbrace u \in U | v(u) \leq \beta \rbrace \end{aligned} $$

The concrete form of *v* is determined by both the specific application and the formal setting used to apply 3WD. It can be, for example, based on conditional probability in the probabilistic rough sets [55], fuzzy neighborhood covering functions [64], dominance relations and their extensions such as variable precision based [51]. The meaning of the three regions is as follows. *POS* is a region of acceptance and includes all objects that can be correctly classified in a specific category. For instance, with reference to the $GS_{t_{0}}$ of Fig. 3, *POS* consists of all the objects that can be classified as SAFE in a particular situation. *NEG* is a region of rejection and includes all objects that can not be correctly classified in a specific category such as, with reference to the $GS_{t_{0}}$ of Fig. 3, all the objects that can be classified as UNSAFE in a particular situation. Lastly, *BND* is a region where the decision has to be deferred. This includes all objects that are dubious with respect to a classification. The advantages of three way decisions are that allow to take rapid decision on tri-classification of a situation such as SAFE, UNSAFE or DOUBT that are simple to understand and aligned to cognitive decision making mechanism of human operators. The three regions represent, de-facto, a further granulation able to provide an additional support, better contextualized to the goal analysis, for the cognitive processes of the decision maker who can use only one of the two granular structures (e.g., Section 5.1) or both (e.g., Section 5.3) to accomplish her task. Figure 4 shows two situations in which: i) the decision maker reasons only with the support of the first granular structure, and ii) the decision maker reasons on both the two granular structures. In the proposed methods it is possible to find also a third case in which the decision maker reasons only on the second granular structure.

Before concluding, we highlight that also other structures can be adopted for situation modeling at the level 2. It is the case, for instance, of lattices of partitions constructed with Formal Concept Analysis and GrC, such as the one we investigated in [20].

### SA Level 3: Projection

In the projection phase, the human operator has to predict evolution of situations in the near future. At this level, the (1) can be as follows: *SitMod*_*L*3_ =< {*GS*(*R*_*j*_)}_*i*_,*NI*,*OS* > with *i* ∈{*γ*,2*γ*,...} refers to a discrete time and *j* ∈{*R*_*B*_,*R*_*C*_,...} refers to a family of binary relations over different subsets of attributes. The requirements to set *i* and *j* are derived from the GDTA.

So, in other words, at this level we employ a family of *GS* derived for different time instants,

that can be constructed according to different binary relations over different subsets of attributes. It may happens, in fact, that, for the projection phase, the SA level 3 requirements indicate a different way of merging information than those of level 2. So, for example, a situation that can be well understood at level 2 in terms of equivalence between objects needs to be projected into the immediate future according to a requirement of spatial proximity. This requires a change to the granulation which must be based on a different binary relation and different attributes.

Now we have to answer why, in determining (1) for level 3, we make projections only on the *GS* and not on the enrichment structures, i.e. *NI* and *OS*. The reason is, first of all, that their modification is not strictly necessary. For example, for the projection phase, we can use an indicator or a signature of another object different from that used in the comprehension phase. But that doesn’t always require an update of the relational or behavioral view to level 1. We remind you that these structures must be designed on the basis of the requirements analyzed by GDTA. Anyway, it is necessary to update data and information on the basis of the projected granular structures, there is a feedback mechanism that informs the previous levels.

## Reasoning on DRB model for intelligence analysis

This section presents some methods and techniques that our research group has developed to enforce Intelligence analysis with the DRB (Descriptive, Relational and Behavioral) Model of situations. Specifically, the methods and techniques are such to support the so-called *analytic tradecraft*. Analytic tradecraft refers to a set of analytic techniques used for Intelligence activities, such as the structured analytic techniques proposed by Heuer [23]. These techniques allow to externalize internal thought processes so that they can be shared and analyzed also by other analysts. The CIA Tradecraft Primer^2^ divides the structured analytic techniques in diagnostic, contrarian, and imaginative thinking: “*diagnostic techniques are aimed at making analytic arguments, assumptions, or Intelligence gaps more transparent; contrarian techniques explicitly challenge current thinking; and imaginative thinking techniques aim at developing new insights, different perspectives and/or develop alternative outcomes*” [39].

These techniques are intended to improve Intelligence analysis by checking the two canonical sources of error: systematic biases and random noise [3] but, in several situations, to obtain these benefits some extensions, such as establishing explicit rules to weight and categorize evidence or incorporating probability theory, can help.

The methods and techniques described in this section are in line with these researches aimed at defining extensions or supports to the analytic techniques. Specifically, the adoption of DRB provides two types of support: structured analytic techniques benefit from *i)* a GrC-based information processing approach that allows information to be granulated according to different perspectives, and from *ii)* the possibility of applying the techniques in an already structured context such as that one of the situation awareness.

Before entering into details, let us give an overview in Table 1 that summarizes for each method: the approaches followed in the modeling phase, how the reasoning is related to analytic techniques for Intelligence analysis, and some applications. The reasoning column refers to structured analytic techniques described in [39].

**Table 1:** Overview of methods and techniques

	Situation modeling	Reasoning
Analysis and reasoning on phenomena in large scale systems. Applications: Epidemic spreading [19] Resilience Analysis [17] Terrorist group identifications [31]	GS induced by binary relations and by evaluation functions enforced with Graph theory measures. 3WD to classify situations.	Diagnostic: making assumption on a phenomenon more transparent. Imaginative: supporting multiple way a situation can develop or evolve (e.g., Alternative Future).
Analysis and reasoning on situations that requires the comprehension of humans or agents behaviors. Applications: Assessing intentional attacks of terrorist groups [18]	Fuzzy signatures are used to model humans or agent behaviors. Signatures are aggregated and compared to comprehend situations. Approaches based on 3WD to support comprehension.	Diagnostic: checking hypotheses and evidences (e.g., Analysis of Competing Hypotheses). Contrarian: high impact / low probability analysis.
Analysis and reasoning on the evolution of situations to understand what could happen. Applications: Detection of anomalous situations [8]	GS induced by equivalence relations. Sequential 3WD to project situations. Similarity measures on GS to evaluate changes.	Diagnostic: analysis and evaluation of different situations (e.g., Analysis of Competing Hypotheses). Contrarian: what if analysis. Imaginative: supporting identification of forces, factors, and trends that would change the situation (e.g., Outside-In Thinking)
Analysis and reasoning on contradictory or opposite situations. Applications: Understanding changes in communities opinions	GS revised in the form of structures of opposition induced by 3WD regions.	Diagnostic: analysis and evaluation of contradictory situations (e.g., Analysis of Competing Hypotheses). Contrarian: reasoning on two contrasting assumptions (e.g., Team A / Team B).

The first method detailed in Section 4.1 is focused on improving the awareness of situations related to the analysis of phenomena involving large scale systems or, in general, systems that can be modeled with graphs. In these cases, it is adopted a combination of techniques based on granulation induced by binary relations and partitions created with evaluation based 3WD, where the evaluation function also includes networks measures to estimate criticality and influence of the nodes of a system. This method enforces reasoning mechanisms that can support diagnostic techniques, supporting analysts in the classification of events and phenomena in a clear way and with a reduced cognitive effort (thanks to 3wD), and imaginary techniques, such as the Alternative Future, with the projection phase.

The second method detailed in Section 4.2 is devoted to the analysis of situations involving humans and/or agents behaviors. In this case, the idea behind the method is to gain a better awareness of the behaviors by employing a fuzzy signature and operations on fuzzy signatures, such as similarity / dissimilarity and aggregation to create groups. 3WD based on probabilistic rough set is, then, used to classify events associated to these behaviors. This method can support reasoning based on Analysis of Competing Hypotheses (that is a diagnostic technique) and High Impact / Low Probability analysis (that is a contrarian technique).

The third method detailed in Section 4.3 aims to support What-If analysis with sequential three way decisions. What-if analysis is performed with the creation of different *GS* reflecting changes in the observations derived from the environment, and with the adoption of similarity measures to compare the *GS*. This method can support several types of reasoning such as outside-in thinking that is based on the identification of forces, factors and trends that would produced changes leading to issues or problems.

The last method proposed in this paper is detailed in Section 4.4. This method leverages on the adoption of probabilistic rough sets to induce structures of opposition, such as hexagons of opposition. The analyst can use this method to evaluate and reason on contradictory or contrary assumptions, and to better understand facts that support changes of opinions in teams and communities.

We observe that the first and the second methods are based on the enforcement of GS at descriptive level with, respectively, Graph theory at the relational level and Object Signatures at the behavioral level. The third and fourth rely exclusively on the descriptive level of our situation model.

### Analysis and reasoning on phenomena in large scale systems

Scenarios, in which large scale systems (e.g., critical infrastructures, geographical regions, etc.) are involved in complex phenomena that have to be analyzed to support tasks of decision makers, require models and algorithms to consider the characteristics as well as the interactions of the components of such systems. Therefore, the aforementioned scenarios foresee the representation of situations according to both descriptive and relational perspectives. In particular, it is possible to build a relational perspective by employing graph theory, i.e., modeling the system components as a graph and a descriptive perspective by using a traditional information table.

More in detail, at SA level 1 (perception), the system to analyze consists of a universe of components *U* and is modeled by considering an information table *IT* = (*U*,*A*) where *A* is the set of attributes describing characteristics of the objects in *U* and by a direct graph *G* = (*V*,*E*), where $V \subseteq U$ is the set of nodes, representing the system components, and *E* is the set of edges (*u*,*v*), where *u*,*v* ∈ *V*. A given edge (*u*,*v*) can model, for instance, a channel allowing a flow of information or physical things from *u* to *v*. Edges can come with a weight $d: V^{2} \rightarrow [0, 1]$ to indicate, for instance, some channel property related to the flow it sustains. In such a representation, *IT* satisfies the descriptive perspective and *G* satisfies the relational perspective.

Moreover, at SA level 2 (comprehension), the processing step is aimed at providing a further level of abstraction about the situation occurring for the monitored system. Thus, the situation comprehension is achieved by synergistically processing both the perspectives coming from SA level 1. In particular, the objective is to evaluate the state of affairs of each component (of the system) by considering their characteristics (from the information table *IT*) and their relations with the other components (the graph *G*). The idea underlying the aforementioned approach is that the situation of a given component is not only function of its own characteristics but even of its neighbors’ situations.

One of the possible ways to implement such aspect is to adopt the Katz Centrality measure [26] and to deploy it into the the context of interest to assess the situation in which a given component is. In particular, if the interest is on the component *u*, its situation can be assessed by using the value obtained by means of the following equation:

7 $$  T(u) = \gamma \sum\limits_{}^{j}d(u, j)T(j) + \phi_{u} $$

where *d*(*u*,*j*) is the weight of the edge (*u*,*j*), *T*(*j*) is the evaluation of node *j*, *ϕ*_*u*_ is calculated by using values coming from the characteristics of *v* and *u* (the information table *IT* and a subset $B \subseteq A$ can be considered). Lastly, *γ* ∈ [0,1] is a balance parameter. In other words, *γ* is able to assign more or less importance to the descriptive or the relational perspective. For example, if *γ* is high then the situation assessment process, for the components, gives more importance to the relations of such components with its neighbors. Otherwise, if *γ* is low then the the aforementioned process will give more importance to the inner characteristics of the component. Now that the individual components’ situations have been assessed, a decision model (see Fig. 4) has to be used to assess the situation of the whole system according to the situations recognized for its individual components. Such model should be able to support the decision makers’ tasks. The proposed decision model is the Evaluation-based Three-Way Decisions [56] that is able to classify all the system components into three main regions, which typically represents positive, boundary and negative regions with respect to a given concept (that is strictly related to the main goal of the decision maker). According to Fig. 2. The three-way decisions is the cognitive tool by which decision makers can receive an assisted view on the situation comprehension process results. More formally, if the focus is on the components criticality *C* (e.g., security, safety), the three regions must be defined according to (8) of Three-Way Decisions theory,

where *ν* represents the *evaluation function* and reports the criticality value of a system component. Such value must be interpreted by using two thresholds: *β* and *α*. In the proposed approach, the evaluation function *ν* addresses a component at a time, whilst *β* and *α* aggregate values coming from all the considered components.

Lastly, SA level 3 (projection) can be useful to estimate the effect of new actions (e.g., increasing the security level of some specific component, limiting the data exchange between two components, etc.) applied on the system. In the proposed approach, the effect of actions is modeled by a specialized function, namely $CA: U \rightarrow [0, 1]$ that is used to modify the evaluation function and/or the thresholds in a way that it is possible to assess the situation after having simulated the application of a given course of actions on the system. Thus, the obtained system situation will take care also of the possible effects of a set of plausible actions applied on it. It is clear that the function *CA* is component-specific, therefore, its values can vary from component to component.

### Analysis and reasoning on situations that requires the comprehension of humans or agents behaviors

To support analysis and reasoning about situations involving groups of humans and/or agents, DRB model leverages on the creation of fuzzy user signatures for such groups. Representing the behavior of such groups in a way that is computationally tractable (such as the adoption of signatures) is crucial in many operational scenarios, such as counter-terrorism and organized crime investigations, where the analysis and evaluation of hypotheses involving humans and groups behaviors is critical to increase situational awareness. If, on the one hand, structured analytic techniques offer numerous diagnostic, contrarian, and imaginative methods of analysis (such as the analysis of competitive hypotheses or the analysis of high impact / low probability scenarios), it remains, on the other hand, a difficulty in correctly deriving the hypotheses and scenarios to be analyzed when this involves human behaviors, such as the terrorist phenomenon. In our vision, the concept of fuzzy user signature, originally developed by Yager and Reformat [50] to represent user’s interests and opinions based on used items and tags, has been adapted to model the behavioral perspective of (1). Let us describe how to adapt this concept to our purposes.

Let us consider an object, *o*, that can perform an action, *a*, with a resource, *r*, on a target, *t*. Examples of objects are humans, software agents and their groups. In counter-terrorism analysis, we focus on considering groups of humans, such as terrorist groups, and understanding their behaviors. A target can be an object or other elements of the environment that can not perform actions (such as bridges, streets, and so on). At SA level 1, the behavior of such objects can be described as a series of temporally distributed events represented as in the following data structure of Table 2 based on categorical values:

**Table 2:** Event dataset

	*time*	*a*	*t*	*r*	*o*
e1	*δ*_1_	*a*_3_	*t*_2_	*r*_2_	*o*_1_
e2	*δ*_2_	*a*_2_	*t*_4_	*r*_3_	*o*_1_
e3	*δ*_3_	*a*_3_	*t*_2_	*r*_2_	*o*_1_
e4	*δ*_1_	*a*_3_	*t*_4_	*r*_3_	*o*_2_
e5	*δ*_2_	*a*_3_	*t*_2_	*r*_1_	*o*_2_
e6	*δ*_3_	*a*_2_	*t*_1_	*r*_3_	*o*_2_

The first row reads as follow: *o*_1_ has performed the action *a*_3_ towards the target *t*_2_ using the resource *r*_2_ at time *δ*_1_. At SA level 2, to better comprehend the behavior of an object, we construct its fuzzy signature following (4). Let us see how to construct the fuzzy sets involved in (4).

Given an object, *o*_*i*_, and a time window, [*δ*_1_,*δ*_3_], a fuzzy set $\overbrace {Act_{o_{i}}}(a)$ can be defined as: $\overbrace {Act_{o_{i}}}(a) = \lbrace \frac {\mu _{1}}{a_{1}}, \frac {\mu _{2}}{a_{2}}, ..., \frac {\mu _{n}}{a_{n}} \rbrace $ with $\mu _{j} = \frac {\# of a_{j}} {|Act|}$ where *j* = 1,2,...,*n* with *n* equal to the total number of actions, and |*Act*| is the cardinally of the set of actions. The membership *μ*_*j*_ is, in other words, the relative frequency of the action *a*_*j*_. In a similar way, the fuzzy sets $\overbrace {Tar_{o_{i}}}(t)$ and $\overbrace {Res_{o_{i}}}(r)$ are constructed. The object fuzzy signature is a fuzzy relation among these sets, such as (4), and its value for a triple (*a*_*j*_,*t*_*k*_,*r*_*l*_) is evaluated as in (5). With reference to the dataset of Table 2, the value of the object signature for *o*_1_ with respect to the triple (*a*_3_,*t*_2_,*r*_2_) is: $OS_{o_{1}}(a_{3}, t_{2}, r_{2}) = 0.667$.

The value of an object signature can be used as a membership of a co-occurrence of a specific action, target and resource. In this way, fuzzy profiles of the behavior of the objects can be created: $O_{i} = \lbrace \frac {OS_{o_{i}}(a_{j}, t_{k}, r_{l})}{a_{j}t_{k}r_{l}} \rbrace $. With reference to the dataset of Table 2, for instance: $O_{1} = \lbrace \frac {0.667}{a_{3}t_{2}r_{2}}, \frac {0.333}{a_{2}t_{4}r_{3}} \rbrace $ where only the signatures ≠ 0 are included.

The fuzzy profile of an object can be considered as an elementary granule of information. The fuzzy profiles can be aggregated using OWA operators to improve the comprehension of granules belonging to the granular structures of the descriptive model of (1). For example, an interesting OWA operator is that one based on probability distribution [49] that, in addition to the set of *n* values to aggregate, have a set of *n* probabilities *p*_*i*_ s.t. ${\sum }_{i=1}^{n} p_{i} = 1$. This kind of OWA has a parameter, *γ*, that allows to generate values ranging from the min (when $\gamma = \infty $) to the max (when *γ* = 0). This flexibility is useful for derivation of different scenarios as we are going to describe in Section 5.2

### Analysis and reasoning on the evolution of situations to understand what could happen

What-if analysis is a simulation technique useful to understand what can happen if some changes occur in the scenario or situation of interest. This technique is used to perform scenario analysis and, therefore, the objective is to present several alternative developments of a situation (i.e., projections) instead of focusing on a single one. In the proposed vision, alternative future representations are constructed in order to reason on projects. Moreover, sequential 3WD [62] is applied to consider more information if needed to support the decision making process.

The main focus of what-if analysis is to support decision makers in understanding which are the factors that can lead to a change in the situation. So, specifically, what are the conditions under which a situation recognized and classified in a certain way, such as safe, can evolve toward situations classified differently, such as unsafe. This can be done by understanding the current situation and projecting that situation on the basis of different conditions. Let us describe how to model a situation, leaving an example of reasoning in the Section 5.3. With reference to (1), the main structure for this case is the descriptive model that can be enforced with the adoption of distance measures to reason on situations.

Starting from the SA level 1 requirements, a decision system *IS* = 〈*U*,*A*〉 is constructed. *A* = *C* ∪ *D* and *d* ∈ *D* is the decision attribute that is used to classify objects with respect to their states. For the sake of simplicity it will be considered only one attribute *d* belonging to *D*. Values for *d* (i.e., safe or unsafe) and could be calculated by means of heuristics and human operators’ knowledge (thus a degree of uncertainty must be considered). At the SA level 2, it is required to comprehend the overall situation. Having classified all the objects of an environment as, for instance, safe or unsafe, we need to assess the overall situation. To perform this task, we *i)* create granular structures as lattices of partitions, and *ii)* apply 3WD based on probabilistic rough sets [55] to classify the parts.

With respect to point *i)*, let *C* be the set of condition attributes and *L*_*e*_ be the lattice constructed by using the equivalence classes [*x*]_*E*_, with *E* belonging to the sequence of subsets $e: E_{1} \subset E_{2} \subset {\dots } \subset E_{m} \subseteq C$. *L*_*e*_ gives a snapshot of the current situation and support comprehensions thanks to the capability of zooming-in (adding more information to obtain finer granules). The comprehension can be enforced with the application of point *ii)*. Let us define $H \subseteq U$ a target concept, consisting of all the objects that are in a desired situation, e.g. *H* could be, for instance, the concept of *safeness*. With 3WD, it is possible to determine POS, NEG and BND regions for *H* at each level of the sequence *e*. Le be $P(H|[x]_{E}) = \frac {| H \cap [x]_{E} |}{| [x]_{E} |}$, the application of 3WD to the subset *E* belonging to *e* is:

8 $$  \begin{aligned} POS(H)= \lbrace x|x \in U, P(H|[x]_{E}) \geq \alpha \rbrace \\ BND(H)= \lbrace x|x \in U, \beta < P(H|[x]_{E}) < \alpha \rbrace \\ NEG(H)= \lbrace x|x \in U, P(H|[x]_{E}) \leq \beta \rbrace \end{aligned} $$

Thus, situation comprehension ends with the construction of a lattice whose levels are built by considering the partitioning of *U* induced by the subsets of *C*. In order to support the projection phase requested to implement the what-if analysis, it is needed to apply situation comprehension to an updated information table *IT*_1_ of our *IS*. For the sake of simplicity, assume that *IT*_0_ is the information table at time instant 0, *IT*_1_ will be the information table at time instant 1 and the two information tables share the same universe *U* and the same set of attributes *A*. Once applied the above described approach to *IT*_1_, a new lattice ${L^{1}_{e}}$ is constructed. Such lattice represents the situation projected to time instant 1 and it can be compared with the previous one by using a dissimilarity measure [29]:

9 $$ Dis({L^{0}_{e}}, {L^{1}_{e}}) = \frac{1}{|U|}\sum\limits_{i=1}^{|U|}\frac{|{L^{0}_{e}}(x_{i}) \bigtriangleup {L^{1}_{e}}(x_{i})|}{|U|} $$

where $|{L^{0}_{e}}(x_{i}) \bigtriangleup {L^{1}_{e}}(x_{i})|$ is the cardinality of a symmetric difference between the the two families of partitions included in ${L^{0}_{e}}$ and ${L^{1}_{e}}$. The result of such dissimilarity measure can be interpreted as a qualitative indicator related to the situation evolution, i.e. high values indicate a situation drift and low values indicate that the situation is not changed also after the occurrence of changes, at time instant 1, to some object characteristics.

### Analysis and reasoning on contradictory or opposite situations

Some structured analytic techniques require a form of reasoning that relies on conflicting and contradictory assumptions. This type of analysis serves to stimulate the analyst’s current mind-set and explicitly challenge current thinking. To this purpose, the SA model can be enforced with structures of opposition, such as squares and hexagons of opposition.

**Fig. 5:**
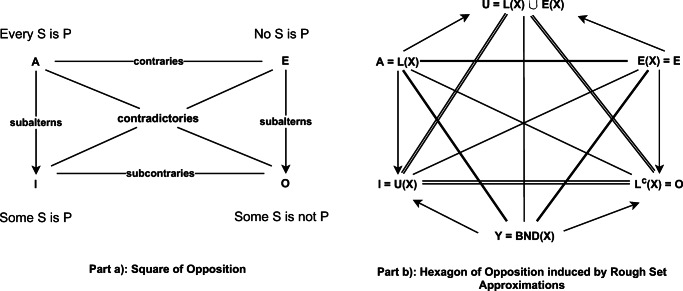
Part a: Square of opposition (From: https://plato.stanford.edu/entries/square/). Part b: Hexagon of opposition indiced by rough set approximations (Elaborated from: [5])

A square of opposition is structure able to show some important laws of Aristotelian logic [33]. The square of opposition is shown in Fig. 5 part a).

In the square of opposition each vertex represents a different statement involving two entities *S* and *P*. Point A and Point E represent, respectively, the universal affirmative and negative and can be expressed as: “Every *S* is *P*” and “No *S* is *P*”. Points I and O represent, respectively, the individual affirmative and negative and can be expressed as: “Some *S* is *P*” and “Some *S* is not *P*”. Clearly, A and I are in opposition to O and E (and vice-versa), A implies I and E implies O. A and E can be false together but not true together, and for I and O it is the converse [5].

Now, let us clarify the connection between structures of opposition and our situation model. The study of structures of opposition has seen the interest of researchers and scholars active in the field of rough sets, fuzzy sets, and orthopairs such as [5, 6, 57, 61] that describe also how to build structures of opposition induced by Rough Sets approximations. A GS, such as that one of the descriptive model, can be represented in terms of a structure of opposition and, thus, can support reasoning and decision making on the basis of contrarian techniques.

We can start from an information table as descriptive model for the SA Level 1. Given an universe *U*, a partition (such as set of equivalence classes [*s*]_*B*_ on a subset of attributes *B*) and a target concept *P* ⊂ *U*, the Pawlack Lower and Upper approximations of *P* are defined as:

10 $$  \begin{aligned} L_{B} (P)= \lbrace s: [s]_{B} \subseteq P \rbrace \\ U_{B} (P) = \lbrace s: [s]_{B} \cap P \neq \emptyset \rbrace \end{aligned} $$

that in terms of regions refer to *POS*(*P*) = *L*_*B*_(*P*), *BND*(*P*) = *U*_*B*_(*P*) − *L*_*B*_(*P*) and *NEG*(*P*) = (*U*_*B*_(*P*))^*c*^. Point A of a square of opposition (i.e., every *s* is *P*) can be mapped into the lower approximation and, similarly, point I (i.e., some *s* is *P*) can be refereed to as the upper approximation. The point E (i.e., no *s* is *P*) is the *NEG*(*P*) = (*U*_*B*_(*P*))^*c*^ (referred to as Exterior region, *E*, in the hexagon of Fig. 5 part b)) and, lastly, point O (i.e., some *s* is *P*^*c*^) refers to (*POS*(*P*))^*c*^ = (*L*_*B*_(*P*))^*c*^.

The hexagon of opposition (see Fig. 5 part b)), or logical hexagon, derives from the square of opposition by adding two other points: the bottom corner of the triangle of contrariety as the conjunction of the I and the O corners and the top corner of the hexagon as the disjunction of the A and E corners. Following the letters chosen by Blanche’ [2], let us refer to Y as the bottom corner and U as the top corner. We have: *Y* = *U*_*B*_(*P*) ∩ (*L*_*B*_(*P*))^*c*^ = (*BND*(*P*) ∪ *POS*(*P*)) ∩ (*BND*(*P*) ∪ *NEG*(*P*)) = *BND*(*P*) and *U* = *L*_*B*_ ∪ (*U*_*B*_)^*c*^ = *POS*(*P*) ∪ *NEG*(*P*).

The six points of the hexagon can be defined by using the three regions:

- A corresponds to the *POS*(*P*)
- E corresponds to the *NEG*(*P*)
- Y corresponds to the *BND*(*P*)
- O corresponds to the (*POS*(*P*))^*c*^ = *NEG*(*P*) ∪ *BND*(*P*)
- I corresponds to the (*NEG*(*P*))^*c*^ = *POS*(*P*) ∪ *BND*(*P*)
- U corresponds to the (*BND*(*P*))^*c*^ = *POS*(*P*) ∪ *NEG*(*P*)

By referring a GS to the points of an hexagon of opposition, the analyst can reason in terms of contradictory or opposite situations as by using only the second level of granulation as described in the example of Fig. 4 resembling, thus, contrarian techniques such as Team A / Team B.

An interesting application of the hexagon of opposition is the structural conceptualism [1] that, basically, is a conceptual analysis devoted to to understand the meaning of concepts by relating them together. In Section 5.4, we show an application of the hexagon of opposition to this kind of conceptual analysis devoted to analyze situations where communities or teams of users change opinions due to new facts and information.

## Case studies

The following subsections report case studies and applications of the methods described in the previous section. Specifically: *i)* Section 5.1 reports an application of the method described in Section 4.1 to analyze the diffusion of COVID-19 in Italy and assess which are the critical regions on the basis of a graph model of Italy and simulating the impact of specific containment actions [19]; *ii)* Section 5.2 reports an application of the behavioral fuzzy profiles for groups described in Section 4.2 for the analysis and assessment of hypotheses on terrorist groups attacks [18]; *iii)* Section 5.3 reports the application of the method described in Section 4.3 to execute what-if analysis for detection of anomalous situations related to surveillance scenarios [8]; *iv)* Section 5.4 reports an application of the adoption of structures of opposition described in Section 4.4 to detect facts and news supporting communities and teams opinion changes.

### Analysis of critical Italian Regions for epidemic diffusion

The approach proposed in Section 4.1 has been implemented to provide a method for estimating, in advance, the effect of containment actions possibly executed by the Italian Government on the Italian Regions to face the diffusion of COVID-19. The idea is to simulate the execution of such actions and evaluate the criticality of the individual Regions once the actions will generate their effects. The simulation can be re-executed after the re-configuration of the actions in a way to build a system able to support the Government decision-making process. Such results have been described in [19].

First of all, Italy is modeled as a graph where nodes represent Regions and edges represent neighborhood relations between couples of Regions. Neighborhood relations can be physical (e.g., between geographical neighbors) or logical (e.g., between Regions connected by significant commercial channels). The weight of the edge (*u*,*j*) is $d(u, j) = \frac {g_{j}}{g_{u}}$, where *g*_*u*_ and *g*_*j*_ are the GPDs (gross domestic product) of the Regions *u* and *j*, which are nodes in the graph. High values (around 1) of *d*(*u*,*j*) mean that there are many people traveling from *u* to *j*. Each node *u* has an additional property, namely *ϕ*_*u*_, representing the population density of the Region. Such graph structures the relational perspective at SA level 1. At the same level, the descriptive perspective is represented by the information system *IS* = (*U*,*A*), where *U* is the set of Italian Regions and *A* is the set of features used to describe each Region. The used features come from the real data (published day by day) about COVID-19 diffusion in Italy provided by the Italian Civil Protection Department. In particular, for each Region the total number of contagions, *TC*(*u*,*t*), total number of executed swabs, *Swabs*(*u*), and number of currently positive people, *AP*(*u*,*t*), have been used. Temporal dependency, in the aforementioned functions, are represented by the variable *t* added as parameter and indicating a specific day.

According to the approach of Section 4.1, at SA level 2, the evaluation function must be applied on each Region to estimate the individual criticality. The evaluation function is defined as $\nu (u, t) = \frac {AP(u,t)}{Swabs(u)}$. Individual criticality have to be interpreted by considering also all the relational aspects.

In fact, in order to assess the criticality of a specific Region, it is needed to consider the criticality values of its neighbors. The idea is to interpret individual criticality by using two thresholds (*β* and *α*) calculated by taking into account not only the data coming from Civil Protection Department but also measures derived by the analysis of the graph modeling the whole Italian system. More in detail, the two thresholds are defined as:

11 $$  \alpha = \vee^{\left | U \right |}_{i=1} (\sigma(u_{i}, t) \wedge \omega(u_{i}, t)), $$

12 $$  \beta = \wedge^{\left | U \right |}_{i=1} (\sigma(u_{i}, t) \vee \omega(u_{i}, t)) . $$

Equations (11) and (12) are respectively the weighted maximum and the weighted minimum. In particular, *α* tends towards the highest *σ*(*u*_*i*_,*t*) that weighs more and *β* tends towards the smallest *σ*(*u*_*i*_,*t*) that weighs less. Moreover, for each Region *u*, $\sigma (u, t) = \frac {TC(u, t)}{Swabs(u)}$ and it can be observed that *ν*(*u*,*t*) ≤ *σ*(*u*,*t*) and 0 ≤ *ν*(*u*,*t*) ≤ 1. Furthermore, the weights *ω*(*u*,*t*) are calculated by using the following formula:

13 $$ \omega(u, t) = CA(u, t) \times T(u). $$

where *T*(*u*) is the criticality value of Region *u* calculated by using the (7) and *CA*(*u*,*t*) ∈ [0,1] measures the freedom degree of each Region. In particular, if *CA*(*u*,*t*) is 1 then the containment actions are not restrictive for Region *u*. Otherwise, if *CA*(*u*,*t*) is 0 then the containment actions are very restrictive for such Region. In other terms, *CA*(*u*,*t*) values represent the way the human decision maker can interact with the simulation (for changing input values for the simulation). Therefore, SA level 2 (comprehension) is completed when for each Italian Region *u*, *ν*(*u*) is positioned in the POS, NEG or BND region by using (6) and thresholds calculated through (11) and (12).

Lastly, at SA level 3 (projection), the values *CA*(*u*,*t*) are of crucial importance since they allow the implementation of a decision support system based on simulations. In other words, the decision maker can establish the *CA*(*u*,*t*) values, representing the containment actions at time *t*, for all Regions basing on her perception and experience. Then, she can get the results of the Three-Way Decisions and assess the situation of the Italian system. If the simulation provides suitable results she can make her decision and choose the current actions. Otherwise, she can increase or decrease *CA*(*u*,*t*) values (discriminating from Region to Region) with respect to her experience and re-execute the simulation. Such simulation helps the decision maker to look for the right trade-off between two objectives: people’s health and country economy.

### Assessment of hypotheses of attack by terrorist groups

This section reports an applications to support counter-terrorism analysis. The analysis executed resembles the structured analytic method called High Impact / Low Probability consisting in the formulation and assessment of different scenarios including those considered to have a low probability of occurrence but which can have a significant impact if they occur.

The overall approach has been described and evaluated in [18]. In the following, the three main phases are highlighted: *i)* evidence collection, *ii)* scenarios derivation and *iii)* scenarios assessment.

The phase of **evidence collection** is devoted at the creation of a body of evidence starting from historical data about terrorist events. This data resembles the event dataset of Table 2 where *e* is a terrorist attack, *a* refers to an attack strategy, *t* refers to a target of an attack, *r* refers to a weapon used in the attack and, lastly, *o* is the decision attribute and refers to the terrorist group that has perpetrated the attack. From this dataset, the three fuzzy sets of (4) can be constructed as explained in Section 4.2 to build a data table of fuzzy classes such as Table 3. A row of Table 3 is a fuzzy model of a group derived from information on attacks, targets, and weapons of a group.

**Table 3:** Fuzzy signature dataset

	a1	a2	a3	t1	t2	t3	t4	r1	r2	r3
o1	0	0,333333	0,666667	0	0,666667	0	0,333333	0	0,666667	0,333333
o2	0	0,333333	0,666667	0,333333	0,333333	0	0,333333	0,333333	0	0,666667

Table 3 can enforce SA Level 1 and 2 since improves the comprehension of how a group behaves. Applying a fuzzy equivalence relation, such as a Gaussian Kernel, to Table 3, a similarity matrix is obtained. Each row of the similarity matrix can be regarded as a fuzzy equivalence class, [*o*_*i*_]. The set of fuzzy equivalence classes is a body of evidence of behaviors’ similarity among different terrorist group. This body of evidence is used to assess attack scenarios, *H*, in the context of a fuzzy probabilistic approximation space [24, 69].

**Fig. 6:**
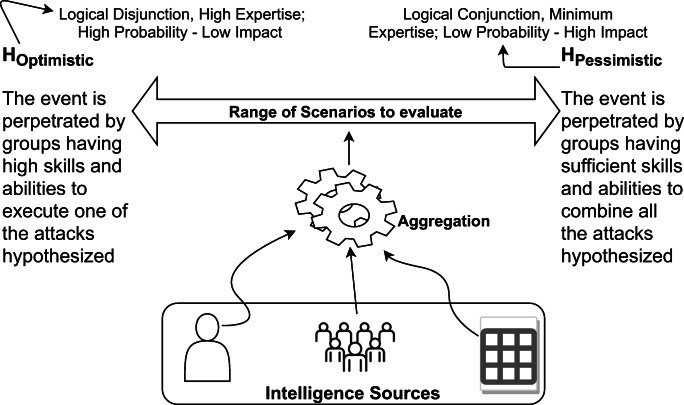
Derivation of Scenarios

The approach to **derive scenarios** for the analysis is shown in Fig. 6. Let us suppose that Intelligence sources provide information on possible attacks. This information is aggregated to derive different scenarios: from Optimistic (refereed to as *H*_*optimistic*_ in Fig. 6) with a high prior probability but low impact (for example, only one out of the *n* attacks is perpetrated) to Pessimistic (refereed to as *H*_*pessimistic*_ in Fig. 6) with a low prior probability but high impact (for example, the *n* attacks are perpetrated together).

Let us call Target Group, *TG*, the potential perpetrator of the attacks hypothesized by Intelligence sources. The objective is to associate *TG* with a known terrorist group. To this purpose, the OWA operator mentioned in Section 4.2 is used to create a profile of the *TG* starting form the object signatures of known terrorist groups:

14 $$ TG = \lbrace \frac{OWA_{i}(OS_{o_{i}}(a_{j}, t_{k}, r_{l}))}{a_{j}t_{k}r_{l}} \rbrace $$

where *i* = 1,2,...,*n* with *n* the number of known groups and $OS_{o_{i}}$ the behavioral signature value of the group *o*_*i*_ for a specific triple (*a*_*j*_,*t*_*k*_,*r*_*l*_).

A scenario of attack, *H*(*TG*), is modeled as a fuzzy event [65]:

15 $$  H(TG) =\frac{\lambda_{1}}{o_{1}} + \frac{\lambda_{2}}{o_{2}} + ... + \frac{\lambda_{n}}{o_{n}} $$

where *n* is the number of known groups, + is the union operator and *λ*_*i*_ is evaluated as [50]:

16 $$  \lambda_{i} = \frac{|o_{i} \cap TG|}{|o_{i}|} $$

Varying the parameter *γ* of the OWA operator, pessimist scenarios can be derived by aggregating the behavioral signatures of known group towards the min, i.e. $H_{pessimistic} = H(TG_{\gamma \longrightarrow \infty })$ and optimist scenarios by aggregating the behavioral signatures of known group towards the max, i.e. *H*_*optimsitic*_ = *H*(*TG*_*γ*→0_). The two situations refer, respectively, to Low probability–High impact and High probability–Low impact scenarios. Furthermore, the introduction of probabilities allows to weight differently groups that are more or less active in a particulate period and geographical area. A probability distribution of the activity of groups is derived by counting the number of events perpetrated by a group refereed to the total number of events.

To improve comprehensions at SA Level 2, the scenarios *H*_*pessimistic*_ and *H*_*optimistic*_ must be **assessed against the body of evidence**. Here, a probabilistic 3WD approach based on a Bayesian Rough Set model is adopted. Given a fuzzy approximation space, a fuzzy event *H*, a pair of thresholds *α* and *β*, and a set of fuzzy equivalence classes [*o*_*i*_]_*R*_, the event can be assessed against the body of evidence using probabilistic 3WD such as (8) where the computation of the conditional probability is defined as follows [69]:

17 $$  Pr(H|[o_{t}]_{R}) = \frac{{\sum}_{i=1}^{n} p(o_{i})r_{ti} H(o_{i})}{{\sum}_{i=1}^{n} p(o_{i})r_{ti}} $$

where *p*(*o*_*i*_) is the probability of *o*_*i*_ and *r*_*ti*_ is the degree of equivalence between *o*_*i*_ and *o*_*t*_. A proper formulation of the thresholds *α* and *β* that is such to deal with low probability events is proposed in [43]:

18 $$  \alpha = \frac{Pr(H)}{Pr(H) + \varepsilon_{1}^ 0 (1-Pr(H))} $$

19 $$  \beta = \frac{{\varepsilon_{0}^{1}} Pr(H)}{{\varepsilon_{0}^{1}} Pr(H) + (1-Pr(H))} $$

where *α* and *β* are function of prior probabilities *Pr*(*H*) and *Pr*(*H*^*c*^), and of two parameters $\varepsilon _{1}^ 0$ and ${\varepsilon _{0}^{1}}$ correlated to the Bayes factor. It is admissible to set $\varepsilon _{1}^ 0 = {\varepsilon _{0}^{1}} = \varepsilon \in [0,1)$ and we can see that if *ε* ≈ 0 then *α* ≈ 1 and *β* ≈ 0, leading to the traditional rough set regions. This implies a strong support of the result by the available evidence. If, instead, *ε* ≈ 1 it leads to the Bayesian rough set model [44] model with *α* ≈ *Pr*(*X*) and *β* ≈ *Pr*(*X*). This implies a weak support of the result from the available evidence. Significance scale values for Bayes factor and *ε* are reported in [43].

With the parameters *γ* and *ε*, it is possible to derive different scenarios (from pessimistic to optimistic) that can be assessed against a body of evidence with different support levels (from very strong to very low). The enforce comprehension and projection at SA Level 2 and 3.

In [18], the method has been evaluated on real data on terrorism events extracted from the GTD^3^.

### Surveillance for detection of anomalous situations

**Fig. 7:**
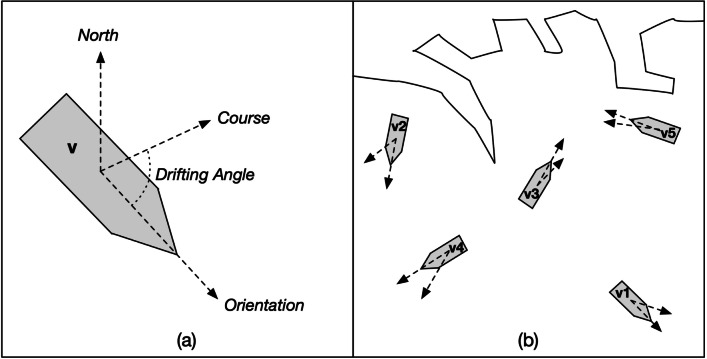
Drifting vessels (elaborated from: [8])

The What-If analysis approach, based on granular computing and situation awareness, described in Section 4.3 has been applied in [8] to a surveillance scenario in the maritime domain. More in detail, such scenario focuses on unsafe situations in which vessels drift and generate possible dangers within the monitored environment. The What-If analysis is used to help operators to anticipate abnormal conditions and to be early warned of possible dangerous situations (near to harbors) by identifying potential drifting vessels while they are docking. Typically, a vessel is said to be drifting when it has a velocity between 3 and 5 knots and an angle between its course and orientation greater than 30 degree (see Fig. 7a). Moreover, Fig. 7b shows a sample scenario that could be analyzed.

Data coming from a specific scenario is organized into a decision table like the one in Table 4 where values can be discretized by using simple IF-THEN rules built on the basis of human experts’ knowledge.

**Table 4:** Sample decision table for What-If analysis

	Velocity	Drifting angle	Distance from coast	Type	Decision (safe or dangerous)
v1	LOW	LOW	FAR	cargo	S
v2	LOW	MID	NEAR	ferry	D
v3	MID	LOW	MID	cargo	S
v4	MID	MID	MID	research	S
v5	MID	LOW	FAR	research	S

**Fig. 8:**
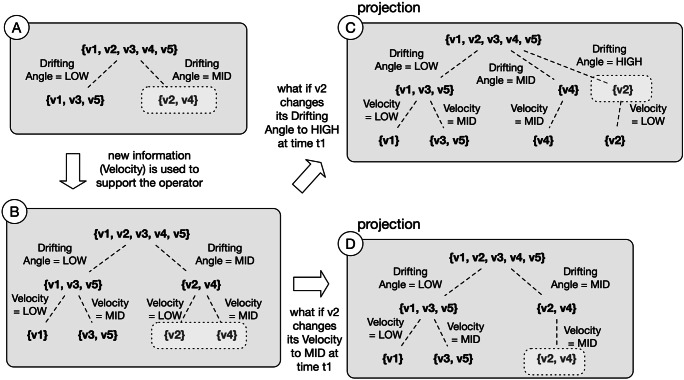
Situation comprehension and projection for drifting vessels (elaborated from: [8])

The values for the *Decision* column are obtained by applying the simple heuristics described before. Once the approach proposed in Section 4.3 has been applied over the decision table provided in Table 4, the results depicted in Fig. 8 are provided as the starting point for the operator’s reasoning process.

Firstly, consider lattice A that is the result of the granulation taking into account the subset of attributes {*DriftingAngle*}. Granule {*v*_2_,*v*_4_} needs attention because its *DriftingAngle* is MID but the operator has to ask for more information to clearly comprehend the situation. Therefore, lattice B is constructed with the subset {*DriftingAngle*,*V* *elocity*}. Granule {*v*_2_,*v*_4_} is decomposed into two granules {*v*_2_} (with LOW velocity) and {*v*_4_} (with MID velocity). The operator can, now, apply her knowledge to understand that *v*_2_ is a potential drifter given that it has LOW velocity and a drifting angle equal to MID. More formally, the concept *S* = {*v*_1_,*v*_3_,*v*_4_,*v*_5_} is constructed to represent *safeness*, *α* = 0.63 and *β* = 0.25 and the only available knowledge is that related to *DriftingAngle*. In this configuration, *P*(*S*|{*v*_1_,*v*_3_,*v*_5_}) = 1 and *P*(*S*|{*v*_1_,*v*_3_,*v*_5_}) = 1, thus *POS*(*S*) = {*v*_1_,*v*_3_,*v*_5_}, *NEG*(*S*) = *∅* and *BND*(*S*) = {*v*_2_,*v*4}. When an additional attribute is considered (i.e., *V* *elocity*), it is possible to calculate *P*(*S*|{*v*_1_} = 1, *P*(*S*|{*v*_3_,*v*_5_} = 1, *P*(*S*|{*v*_2_} = 0, *P*(*S*|{*v*_4_} = 1, thus *POS*(*S*) = {*v*_1_,*v*_3_,*v*_4_,*v*_5_}, *BND*(*S*) = *∅* and *NEG*(*S*) = {*v*_2_}. A substantial shift from situation represented by lattice B and situation represented by lattice C can be measured by considering the (9). In particular the distance is *Dis*(*L*^*B*^,*L*^*C*^) = 0.2. Secondly, if it is needed to reason on the situation projection, simulation scenarios have to be constructed. In this example, two scenarios will be described. In particular, in the first scenario, the operator wants to project a situation in which the *DriftingAngle* of *v*_2_ is assumed to change its value to HIGH (increase). Hence, a new decision table is constructed and a new lattice is provided by using a granulation over two levels {*DriftingAngle*}⊂{*DriftingAngle*,*V* *elocity*}. Lattice C reports the corresponding situation representation. If observing lattice B and lattice C it is possible to conclude in advance (only by considering *DriftingAngle* without adding the *V* *elocity* attribute) that *v*_2_ is dangerous. A second possible projection scenario is explored by the operator by increasing the *V* *elocity* of *v*_2_ from LOW to MID. In this case, the lattice D is obtained. If comparing lattice B and lattice D it is possible to conclude that the situation of *v*_2_ is safer when its velocity increases. In fact, *P*(*S*|{*v*_2_,*v*_4_}) = 0.5 in lattice D, and *P*(*S*|{*v*_2_}) = 0 and *P*(*S*|{*v*_4_}) = 1 in lattice B, when considering the same subset {*DriftingAngle*,*V* *elocity*}.

### Analysis of communities opinions changes

The hexagon of opposition induced by rough set approximations can be adopted to identify news that support opinion changes in communities. A community is a set of users that express an opinion or a judgment on the news. With reference to the approach described in Section 4.4, if the target concept consists of a community of users having positive opinions, point A includes the communities having positive opinions on the set of news, point E includes the communities with negative opinions and point Y includes the communities without a clear positive or negative opinion. Point I consists of communities where the majority of users have not negative opinions (i.e. they are undecided or have a positive opinion), Point O consists of communities where the majority of users have not positive opinions (i.e. they are undecided or have a negative opinion) and point U includes the communities with a clear positive or negative opinion. The idea behind the proposed method is to build different hexagons of opposition incrementally, as additional news are evaluated by users of the communities and, then, compare the points of the hexagon with a cardinality measure to understand the news that polarize opinions in a positive or negative way.

At SA Level 1, there is an information systems, *IS* = (*U*,*I*), where *U* = {*u*_1_,...,*u*_*n*_} is an universe of users who are described by means of a set of attributes *N* = {*N*_1_,...,*N*_*m*_}. These attributes refer to news evaluated by the users. Let us define a function $I: U \rightarrow V_{n}$ for every *n* ∈ *N* where *V*_*n*_ is the set of values that an attribute may take. *I* provides opinions, believes, sentiments or emotions that can be extracted from text of the users’ comments attached to the news or by other means.

**Fig. 9:**
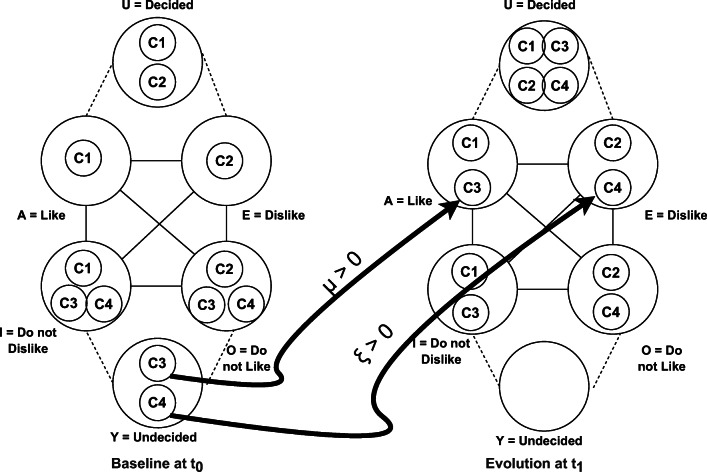
Analysis and comparison of hexagons of opposition

To support comprehension at SA Level 2, two hexagons of opposition are constructed: the first represents a baseline situation and the second reports an evolution. Let $T \subseteq U$ be a target concept to analyze and *C* = {*c*_1_,...,*c*_*q*_} be a partition of *U* in *q* communities. To build the baseline hexagon, let us fix a time, *t*_0_, and a subset $N_{t_{0}} \subseteq N$ of news shared at time *t*_0_. Using a 3WD approach the hexagon of opposition is constructed by following the correspondences among the three regions and the six points as described in Section 4.4. At *t*_1_ > *t*_0_, when additional news, $N_{t_{1}}$ s.t. $N_{t_{1}} \cap N_{t_{0}} = \emptyset $, have been evaluated, another hexagon at *t*_1_ is built. Depending on the specific type of analysis, the update can also lead change in the configuration of the target concept *T* because, for instance, the additional news have changed some opinions. The hexagons at *t*_0_ and *t*_1_ are then compared. Let us look at Fig. 9 that refers to a partition of *U* in 4 communities, *c*_1_,...,*c*_4_.

Figure 9 shows a baseline hexagon built at *t*_0_ and its evolution at time *t*_1_. The communities that change opinions are marked with arrows and two measures, *μ* and *ξ*, are defined to quantify the changes of opinions in the point A “Like” (measure *μ*) and point E “Dislike” (measure *ξ*) points of the hexagons. Specifically, Fig. 9 shows an increase of the decided users with clear opinions (positive or negative). This can be observed from the fact that communities *c*_3_ and *c*_2_ moved, respectively, from point Y to point A and from point Y to point B. The news produced a polarization around positive opinions for the community *c*_3_ and around negative opinions for *c*_2_. The two measures are, in this case, greater than 0. Other cases, not shown in Fig. 9, can occur with different combinations of *μ* and *ξ*, such as polarization of communities around doubts or negative opinions (*μ* < 0 and *ξ* > 0), or increase of indecision (*μ* < 0 and *ξ* < 0).

A concrete implementation of *μ* and *ξ* can be formulated on the basis of the gain measures of Game Theoretic Rough Sets [22].

## Conclusions and open issues

The main result presented in this paper is a model, namely DRB model, to represent and reason on situations related to Intelligence analysis activities. The model consists of three complementary perspectives, namely the descriptive, relational and behavioral, and it has been instantiated with four case studies related to the use of structured analytic techniques adopted by the Intelligence community. The model draws its origins from a work of refinement and abstraction of previous research results related to the use of the SA paradigm and of GrC methods and techniques to Intelligence analysis scenarios and presents several distinctive aspects.

Firstly, the proposed model allows to represent situations according to the SA paradigm and, therefore, operational situations in which the information requirements needed for perception, comprehension and projection (Endsley’s Model of Situation Awareness) are defined in relation to goals and tasks in a structure called GDTA which, by its intrinsic nature, supports analysts with a hierarchical decomposition of information facilitating decision-making. Secondly, it leverages the principles of GrC and 3WD to process information and support rapid decision-making with reduced cognitive effort, biases and other factors weakening situational awareness and compromising the quality of decision-making. All such aspects are fundamental for Intelligence analysis. Thirdly, the model has the characteristic of being actionable and allows a computational treatment of complex aspects such as the modeling of behaviors and relationships between human and/or software agents. As an example, the use of fuzzy user signatures to model human behavior in counter-terrorism scenarios has proved to be a useful support to enforce the descriptive perspective of the situation.

The aforementioned characteristics make the proposed model a useful cognitive support to the Intelligence communities where the need for maintaining the Human-in-the-Loop and improving the response time of decision-making processes are crucial.

The studies also revealed some open issues of a methodological and technological nature. With regards to the former, some open issues concern the modeling of situations involving groups of human and software agents. The current approach based on fuzzy signatures allows the use of aggregation operators, such as OWA, to compute the behavior of groups. However, the challenges related to a more precise modeling of this aspect and those related to its computational treatment remain still open. Their resolution is necessary for the analysis of human phenomena, such as terrorism, and requires a study aimed at modeling cognitive and semantic processes underlying social behavior. On the other hand, the open issues relating to the definition of methods and tools to support the assessment of evolving situations could be challenged from both methodological and technological viewpoints. In particular, with respect to the present work, such issues translate into the definition and development of techniques for the creation and evolution of granular structures, such as rough set partitions, starting incrementally from the data streams produced by sensors deployed into large environments. Moreover, the adoption of the proposed model within OSINT (Open Source INtelligence) applications represents a further noteworthy perspective of research.

Lastly, the DRB model represents the basis for new developments in terms of a technological framework based on Big Data technologies, such as Apache Spark^4^ and Apache Kafka^5^, which is currently under development and supports the creation of rough sets approximations and, consequently, the application of the 3WD on data streams incrementally, as time and data flow.

## Footnotes

1 https://nso.nato.int/natoterm/Web.mvc

2 https://www.hsdl.org/?abstract&did=20945

3 https://www.start.umd.edu/gtd/

4 https://spark.apache.org/

5 https://kafka.apache.org/
